# Roll eccentricity extraction and compensation based on MPSO-WTD and ITD

**DOI:** 10.1371/journal.pone.0259810

**Published:** 2022-02-25

**Authors:** Shanfeng Gao, Lei Xu, Yongkang Li, Jiwen Ji

**Affiliations:** 1 School of Automation and Software Engineering, Shanxi University, Taiyuan, Shanxi, China; 2 College of Mechanical and Vehicle Engineering, Taiyuan University of Technology, Taiyuan, Shanxi, China; Torrens University Australia, AUSTRALIA

## Abstract

To meet the high thickness accuracy requirements in cold-rolling processes, a roll eccentricity signal extraction method based on modified particle swarm optimization and wavelet threshold denoising (MPSO-WTD) with intrinsic time-scale decomposition (ITD) is proposed. The strong denoising ability of the wavelet is combined with the decomposition and recognition attributes of ITD for non-stationary signals. Periodic disturbances in strip thickness caused by roll eccentricity are actively compensated. First, the wavelet is used to denoise the signal and the MPSO algorithm is applied to determine a rational threshold and improve the calculation efficiency. Then, the denoised signal is decomposed into proper rotational components (PRCs) using the ITD method, and an appropriate PRC component representing the eccentricity signal is extracted. Finally, the eccentricity compensation signal is applied in the automatic gauge control (AGC) system of the cold rolling mill. During the rolling process, the rolling speed is not constant and will directly affect the frequency of the roll eccentricity signal. To solve this problem, an encoder is installed at the end of the roll and the compensation frequency of the roller eccentricity signal is determined in the roller eccentricity compensation system according to the pulse number output. The results of simulations and experiments show that roll eccentricity signals extracted using the proposed method can effectively remove the influence of interference signals. An average improvement of 62.3% in the roll eccentricity compensation effect was achieved under the stable rolling condition in the finishing rolling stage.

## 1. Introduction

In strip production, aluminum alloy strip quality is one of the most important factors affecting consumer selection when deciding among similar competing products. Strip quality is mainly assessed by thickness error and flatness error. In the rolling process, many factors can affect strip quality by causing deviation in thickness or defects, and roll eccentricity is a key factor [1]. Roll eccentricity often exists in strip rolling processes, and is generally caused by inexact roll grinding, work roll, or back-up roll ovality [2].

The existence of roll eccentricity can lead to periodic fluctuations in rolling force and roll gap and may adversely affect the control effects of traditional automatic gauge control (AGC) systems [3]. Thus, roll eccentricity compensation is essential. Accurate compensation relies on accurately extracting the eccentricity signal, which can be affected by a variety of disturbance signals.

At present, there are a variety of methods for analyzing roll eccentricity signals, such as neural network prediction methods [4–7], fast Fourier transform (FFT) and modified FFT (MFFT) algorithms [8–10], and wavelet transforms. A neural network-based technique was previously presented for identifying three key factors of roll eccentricity based on the measured angular velocity of rolls [11]. However, only the fundamental wave can be identified leading to limited compensation accuracy. Rolling mill stands without angular velocity sensors are common due to cost constrains and economic considerations [12]. Furthermore, large amounts of data are required to train the models, which places high demands on hardware systems resulting in low calculation efficiency [3]. The Fourier transform is widely used to process linear stationary signals and can be used to effectively analyze the frequency characteristics of signals. However, the method is not suitable for processing local signal information and introduces distortion in denoising nonlinear and non-stationary signals [13–15]. Although the difference evolution algorithm used in the MFFT removes some restrictions of the traditional Fourier transform computational efficiency is reduced [12].

In contrast to the FFT, wavelet transform analysis offers good localization characteristics in both the time and frequency domains [16]. Moreover, the wavelet transform is not restricted by sampling duration requirements or influenced by signal acquisition noise, and has therefore been widely used in denoising operations. The wavelet threshold denoising method can conveniently and flexibly extract roll eccentricity signals. However, it should be noted that frequency aliasing and redundant images can emerge during wavelet decomposition and reconstruction process [17], therefore, the method does not guarantee that roll eccentricity components will reflects the real situation in rolling mills.

Many scholars have attempted to overcome the frequency aliasing phenomenon in wavelet decomposition using demodulation methods or combining wavelet denoising with various algorithms. Demodulation methods include generalized demodulation signal decomposition [18], iterative generalized demodulation [19], and parameter resolution modulation [20]. Frequency demodulation methods can eliminate cross interference among signal components under certain conditions, however, instantaneously intersecting frequency signal components cannot be separated. An eccentricity signal extraction method combining improved wavelet denoising and ensemble empirical mode decomposition (EEMD) was previously proposed [3]. The EEMD method can suppress the frequency aliasing phenomenon of the wavelet algorithm and improve the eccentric signal extraction accuracy. However, when dealing with time-varying non-stationary signals, the number of signal components obtained by EEMD is usually larger than the actual number of characteristic components, therefore, false components with no correlation to the signal characteristics may arise, and calculation efficiency is low [21].

The intrinsic time-scale decomposition (ITD) method can overcome spectral aliasing and has high computational efficiency and precision, however, its noise resistance is poor [21]. At present, the ITD method is mainly used in the field of mechanical fault diagnosis for diagnosing gear faults [22] and diesel engine faults [23]. Previous research has demonstrated high accuracy of the ITD method in signal feature extraction.

Roll eccentricity is not constant in rolling process. For instance, the threading situation as well as speed up/down situation, the rolling speed varies quickly, so as to the change of eccentricity frequency, and the eccentricity amplitude will also vary due to the abrasion of rolls [12]. To improve the accuracy of roller eccentricity signal compensation, the influence of rolling speed and roll wear on eccentricity signal should be considered.

To improve roll eccentricity signal extraction, this paper proposes an MPSO-WTD method with ITD. The algorithm combines the advantages of wavelet analysis with those of the ITD method. To prevent changes in rolling speed from influencing the roller eccentricity signal compensation system, an encoder is installed at the end of the roll and the compensation frequency of the roller eccentricity signal is controlled according to the pulse number output. Roll wear accumulates slowly and can influence product yield. To further ensure stability of the control system, the eccentricity compensation signal is periodically modified online according to the set rolling production requirement.

Finally, to verify the effect of the eccentricity compensation signal on improving strip thickness characteristics, compensation signals were input into the AGC system of a four-high irreversible cold strip rolling mill.

## 2. MPSO-WTD method

### 2.1 Wavelet threshold function

Denoising methods using wavelet thresholding are based on the assumption that the energy of the useful part of the signal will be concentrated in a small number of large-amplitude coefficients. However, most noise energy is dispersed throughout a large number of small-amplitude coefficients. Based on this fact, wavelet coefficients corresponding to the signal will be greater than the noise after wavelet decomposition. The noise can be suppressed by selecting a suitable threshold and properly processing the wavelet coefficients and the main signal features can be preserved. Thus, the key factors in wavelet thresholding are threshold estimation and construction of the thresholding function [24]. That is, after a suitable threshold is selected, an appropriate thresholding function can be used to compress the wavelet coefficients.

Soft and hard thresholding functions are the most used. Overall discontinuity of hard thresholding functions can lead to abrupt shock points in denoised signals, which is particularly obvious when the noise level is high [25]. When a soft thresholding function is used, there will be some deviation between the estimated wavelet coefficient and the real signal wavelet coefficient [26]. In this paper, a new thresholding function is adopted [24]. The new thresholding function is a compromise between hard thresholding and soft thresholding. The constant deviation between the estimated wavelet coefficient and the wavelet coefficient of noisy signals can be modified by changing the value of the regulation coefficient α. The thresholding function is

$${\hat{\omega }}_{j,k}=\{\omega_{j,k}-\mathrm{sgn}(\omega_{j,k}){\begin{array}{c} \left(\sin\left(\frac{\pi }{2}\cdot \frac{\lambda }{\omega_{j,k}}\right)\right)\\ 0,(|\omega_{j,k}|<\lambda ) \end{array}}^{\alpha }\cdot \lambda ,\}(|\omega_{j,k}|\geq \lambda )$$ (1)

where α is the regulation coefficient and *λ* is the threshold. This new thresholding function has the same continuity as the soft thresholding function in the wavelet domain but approaches the hard thresholding curve as the wavelet coefficients increase [24].

The wavelet denoising effect is improved by selecting the optimal threshold. The new thresholding function in Eq (1) improves the flexibility of the threshold function and allows the wavelet threshold values to be adaptively selected, to a certain extent. In this paper, the Donoho threshold is used and can be expressed as

$$\lambda_{j}=\sigma_{j}\sqrt{2\text{ln}\;n_{j}}$$ (2)

where *λ*_*j*_ is the wavelet threshold of layer j; *n*_*j*_ is the length of the wavelet coefficient on scale j; $\sigma_{j}=MAD(|\omega_{j,k}|,0\leq k\leq 2^{j-1})/q$, MAD(.) is an operator that computes the median value.

The wavelet threshold of each layer can be obtained using the gradient iteration method. Long iteration times and complex characteristics of roll eccentricity signals will reduce the denoising effect. In this paper, the modified particle swarm optimization (MPSO) algorithm is used to search for the optimal threshold and shape adjustment parameters using the root mean square error (RMSE) between the original signal and denoised signal as fitness function.

### 2.2 Wavelet threshold optimized by MPSO

Particle swarm optimization (PSO) has the advantages of a simple concept that is easy to implement and fast convergence. Each particle represents a possible solution to an optimization problem and characteristics of a particle include its fitness value, velocity, and position. The fitness value is calculated using the adaptation function. In each iteration, the particle velocity (V) and position (X) are updated, as follows:

$$V_{id}^{k+1}=\omega V_{id}^{k}+c_{1}r_{1}(P_{id}^{k}-X_{id}^{k})+c_{2}r_{2}(P_{gd}^{k}-X_{id}^{k})$$ (3)

$$X_{id}^{k+1}=X_{id}^{k}+V_{id}^{k+1}$$ (4)

$$\left\{\begin{array}{l} V_{id}=V_{\max}\;V_{id}>V_{\max}\\ V_{id}=-V_{\max}\;V_{id}<-V_{\max} \end{array}\right\}$$ (5)

where ω is the inertia weight; *d* = 1, 2, …, *D*; *i* = 1, 2, …, *n*; *k* is the current number of iterations; *c*_1_ and *c*_2_ are acceleration coefficients; *P*_*id*_ is the best position of the individual particle, *p*_*gd*_ is the optimal position of the whole particle swarm.

The PSO algorithm can easily fall into local optima, resulting in premature convergence. During the solution process, the algorithm considers a variety of information including previous information of the individual, the best position of each particle, and the best position of the total swarm. However, the influence of other individual particle information on particle motion is not considered. Here, a modified particle swarm optimization (MPSO) algorithm is introduced into the threshold determination process [27]. The particle velocity in Formula (3) is updated as follows:

$$V_{id}^{k+1}=\omega V_{id}^{k}+c_{1}r_{1}(P_{nd}^{k}-X_{id}^{k})+c_{2}r_{2}(P_{gd}^{k}-X_{id}^{k})$$ (6)

where $P_{nd}=\frac{1}{n}\sum_{i=1}^{n}P_{id}$ is the average best position of all individual particles.

The main advantage of MPSO is the solution process, which seeks the optimal particle solution. Each particle not only obtains its own optimal position information, but also learns from information about other individual particles in the group. In this way, the particle search direction is determined using more effective information, leading to faster convergence rates. The MPSO algorithm is found to have higher search accuracy and stronger optimization ability, with great improvements in stability and convergence speed compared with the traditional PSO algorithm [28].

In general, the root mean square error (RMSE) between reconstructed signals and original signals is the standard measure of a reconstructed signal quality, expressed as

$$RMSE=\sqrt{\frac{1}{N}{\sum_{i=1}^{N}\left[f(i)-\hat{f(i)}\right]}^{2}}$$ (7)

where *f*(*i*) is the original signal and $\hat{f}\left(i\right)$ is the denoised signal.

According to Eq (1), when threshold λ and regulation coefficient α are selected, the wavelet coefficient of the thresholding function can be obtained; that is, the reconstructed signal can be determined after denoising. Therefore, the vector composed of threshold λ and coefficient α can be regarded as the particle position in the MPSO algorithm and represents a potential solution vector. The corresponding fitness value is obtained using Eq (7).

**Fig 1:**
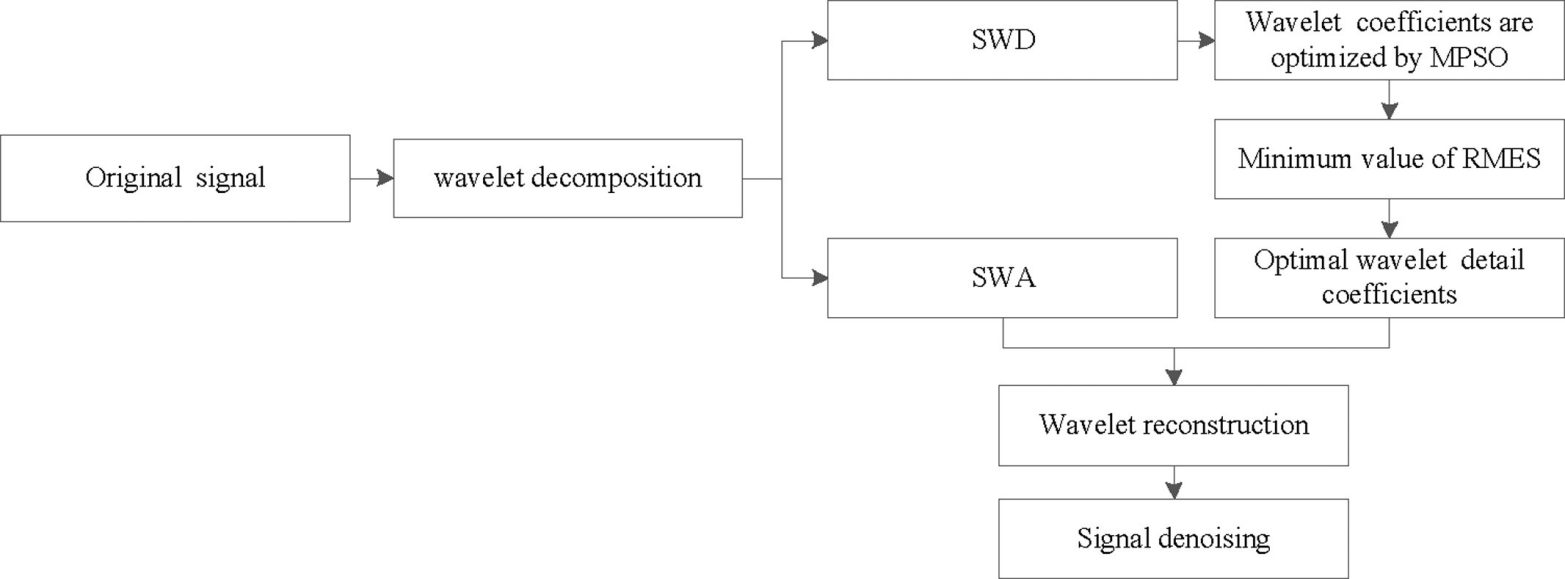
Flow chart of MPSO-WTD algorithm.

The MPSO process for optimizing the wavelet threshold algorithm is illustrated in Fig 1. The specific steps are as follows:

Step 1: Signal preprocessing. Collect the rolling force signal, select the appropriate wavelet basis function, and determine the number of decomposition layers N.
  If the number of decomposition layers is too large, threshold processing of the wavelet spatial coefficients of each layer will cause serious signal information losses, the signal-to-noise (SNR) ratio will be reduced, and the computing speed will be slow. Similarly, an insufficient number of decomposition layers will also affect the final denoising effect. Here, the SNR of the denoised signal is repeatedly calculated using Eq (8) and the number of decomposition layers N is optimal. The SNR is calculated as
$$SNR=10\log_{10}\frac{f(i)}{\hat{f}(i)}$$ (8)
Step 2: Signal decomposition. Decompose the initialized signal to obtain the wavelet coefficients of each layer.
Step 3: Parameter optimization. Optimize the wavelet coefficients of each layer using the MPSO algorithm. The specific method is as follows:
  Initialize the search space and position of the MPSO algorithm. Different thresholds should be set for different layers, in which the particle position consists of threshold λ and regulation coefficient α.
  1. Calculate the fitness value of each particle according to Eq (7).
  2. Use Eqs (4) and (6) to update the velocity and position of the particle.
  3. Update position *P*_*nd*_ as the average of the best position of all particles in the total population and optimal value *P*_*i*_ of the current position of each particle. Compare the new value with the previous optimal value.
  4. Repeat steps (2)-(4). The optimal value of the wavelet threshold of the current layer j is obtained when the termination condition or maximum number of iterations is reached.
Step 4: Signal reconstruction. Use the optimal threshold λ and regulation coefficient α of each decomposition layer to denoise the signal and output the denoised signal.

### 2.3 ITD method

The ITD method is self-adaptive and can decompose complex non-stationary signals into several PRCs and a monotonic trend component. The method calculates the instantaneous frequency and amplitude of each rotation component in each local period using a piecewise method, which can overcome mode aliasing and realize real-time data processing [29, 30]. Thus, the ITD method is suitable for online signal data processing. In addition, the baseline (mean curve) definition is derived through linear transformation of signals, which can shorten the calculation time and reduce error in the fitting process, thereby achieving high calculation efficiency and high calculation accuracy.

The basic steps of ITD are described below, where L is defined as the baseline extraction operator for signal *X*_*t*_.

Defining *L*_*t*_ = *LX*_*t*_, the operator can be used to represent the baseline curve of the signal with *X*_*t*_ = *L*_*t*_ + *H*_*t*_, where H_t_ is defined as a reasonable PRC.

The specific operation process can be described as follows:

1. Determine the extreme value *X*_*k*_ and corresponding time *τ*_*k*_ of signal *X*_*t*_(t ≥ 0), where M is the number of extreme points.
2. Determine extraction operator L of the piecewise linear baseline of the signal:
$$L=L_{k}+(L_{k+1}-L_{k}/L_{k+2}-L_{k})(X_{t}-X_{k}),t\in (\tau_{k},\tau_{k+1})$$ (9)
  with
$$L_{k+1}=\alpha [X_{k}+\left(\frac{\tau_{k+1}-\tau_{k}}{\tau_{k+2}-\tau_{k}}\right)(X_{k+2}-X_{k})]+(1-\alpha )X_{k+1}\;k=1,2,\ldots ,M-2$$
  where 0 < *α* < 1. Normally, α is 0.5.
3. Define the PRCs used to extract the operator.
$$H_{t}^{1}=HX_{t}=X_{t}-LX_{t}=X_{t}-L_{t}^{1}$$ (10)
  where $H_{t}^{1}$ is the PRC component with the highest frequency and baseline signal $L_{t}^{1}$ can be used as the initial signal.
4. Repeat steps (1)-(3) until the baseline signal is a monotone function or a constant function. Then the original signal can be decomposed into
$$\begin{array}{l} X_{t}=LX_{t}+HX=HX_{t}+(H+L)LX_{t}\\ =[H(1+L)+L^{2}]X_{t}=[H\sum_{K=0}^{P-1}L^{K}+L^{P}]X_{t}\\ =H_{t}^{1}+H_{t}^{2}+H_{t}^{3}+\ldots \;H_{t}^{P}+R \end{array}$$ (11)
  where $H_{t}^{P}$ is the *p*th rotation component and R is a monotone function or residual term.

In the signal decomposition process with ITD, the local mean value of the signal is calculated by extracting the local extreme value of the signal. However, the distribution of extreme points of the signal will be affected by environmental noise and the anti-noise performance of the ITD method is poor. To improve the accuracy of signal feature extraction, it is necessary to eliminate noise in the ITD signal as much as possible. Therefore, the MPSO-WTD method is adopted as a denoising pretreatment.

## 3. Simulation experiment

The roll eccentricity signal of a cold rolling mill can be represented by the rolling force signal, roll gap signal, and other signals. Therefore, the roll eccentricity signal can be regarded as a series of superimposed high-frequency sinusoidal periodic waves and a complex signal composed of random noise signals. The frequency depends on the speed of the support roller [3]. Here, the influence of roller thermal deformation and wear on the amplitude of the eccentricity signal is not considered. The eccentricity signal of a four-high non-reversible cold rolling mill is defined as

f(t)=0.03sin(10t+7.3)+0.018sin(9.23t+15.2)+n(t) (12)

where *n*(*t*) is a random noise signal.

**Fig 2:**
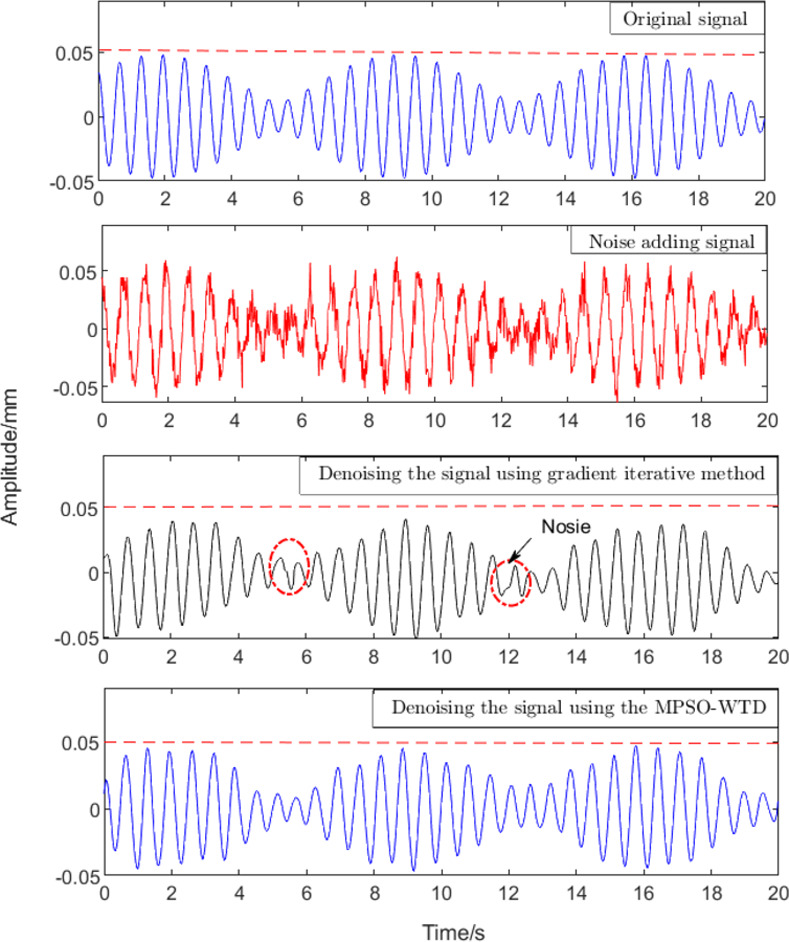
Comparison of signal denoising effect.

In this study, the cofi5 wavelet basis function was used to decompose the signal. The number of decomposition layers was 7 and the signal was decomposed using the above method. The threshold value (λ) and the adjusting parameter (α) were determined using an iterative method and the MPSO algorithm, respectively. The reconstructed signal after denoising is shown in Fig 2. Compared with the gradient iteration-wavelet denoising algorithm, the MPSO-WTD algorithm can better retain the original signal information. The RMSE of the results obtained using the MPSO-WTD algorithm and iterative method were 0.16 and 0.28, respectively, and the corresponding SNRs were 9.13 and 7.24. The results show that MPSO-WTD not only improves the denoising effect of eccentricity signals, but also preserves singularity of the original signal.

**Fig 3:**
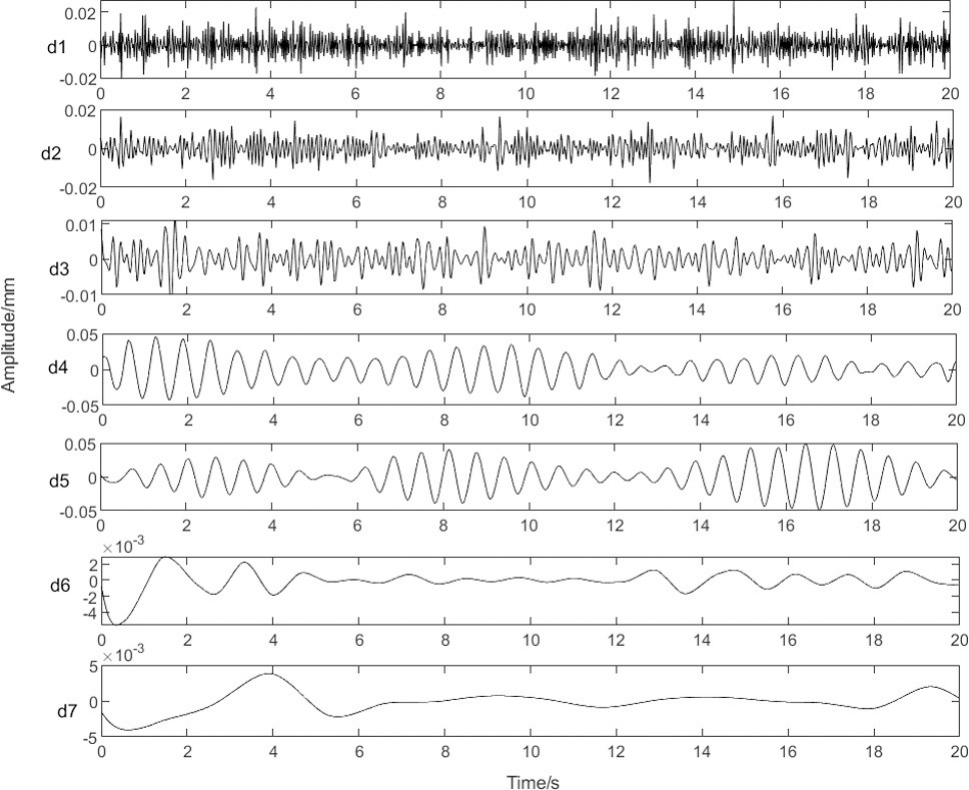
Approximated waveforms using coefficients of each layer of the wavelet decomposition.

**Fig 4:**
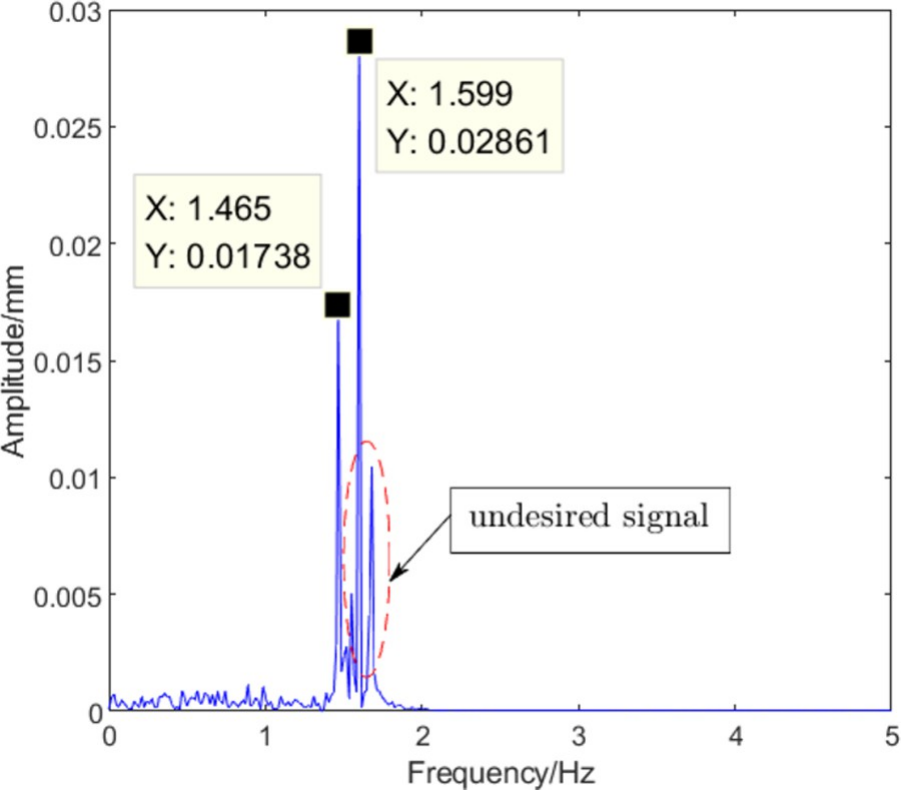
Amplitude-frequency characteristics of d5.

Fig 3 shows approximate waveforms obtained using the coefficients of each layer of the wavelet decomposition. It is known that d5 can reflect the roll eccentricity signal by Fourier analysis. The amplitude-frequency characteristics of d5 are shown in Fig 4. The signal extracted using the wavelet decomposition method has unrelated frequency information, which will result in interference in the reconstructed eccentricity signal.

**Fig 5:**
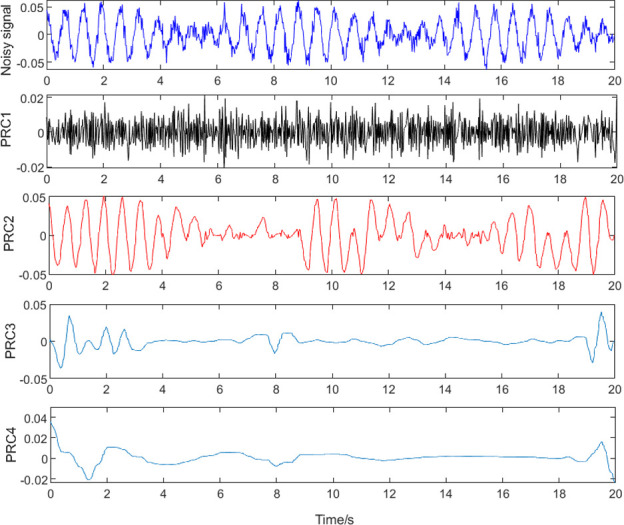
Intrinsic time-scale decomposition (ITD).

**Fig 6:**
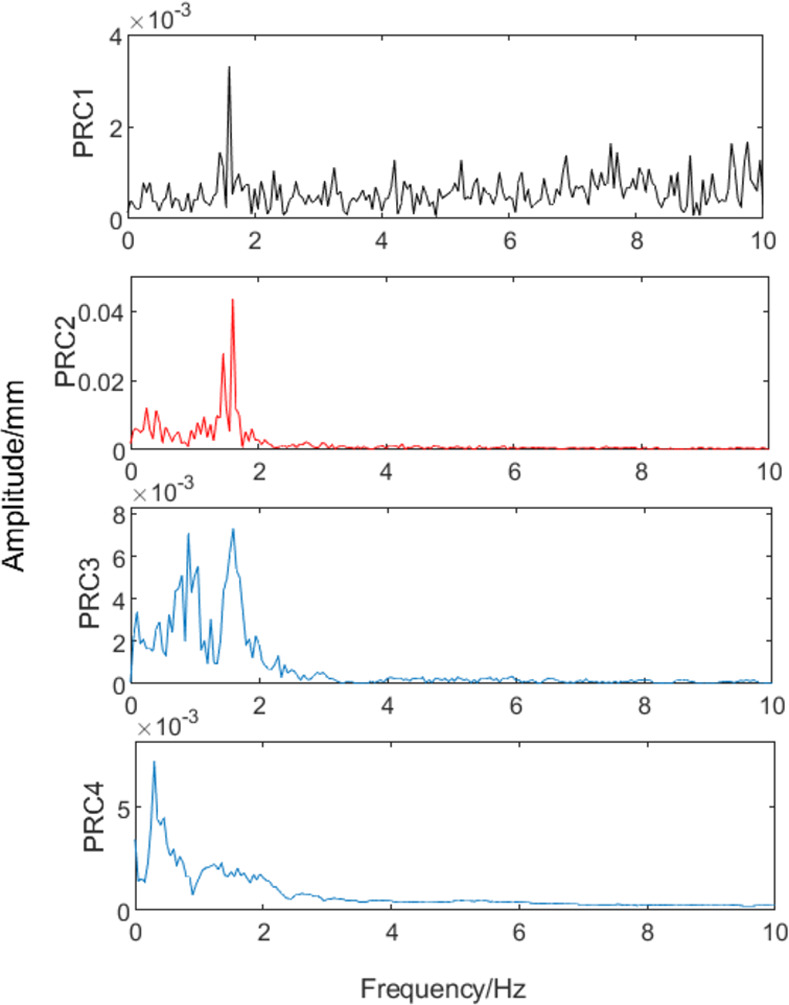
Amplitude-frequency characteristics of each proper rotation component (PRC).

The signal model presented in Eq (12) was decomposed by the ITD method and the result is shown in Fig 5. The amplitude-frequency characteristics of each PRC are shown in Fig 6. While PRC2 contains eccentricity signals of 1.6 Hz and 1.45 Hz, other spurious frequencies can be observed. Moreover, an eccentricity signal with a frequency of 1.6 Hz is present in PRC1 and PRC3. The results show that noise will disturb the distribution of extreme points in the signal in the ITD process; therefore, the accuracy of the calculated results will be affected. To improve the accuracy of signal feature extraction, it is necessary to eliminate the influence of noise signals in eccentricity signal extraction by ITD as much as possible.

**Fig 7:**
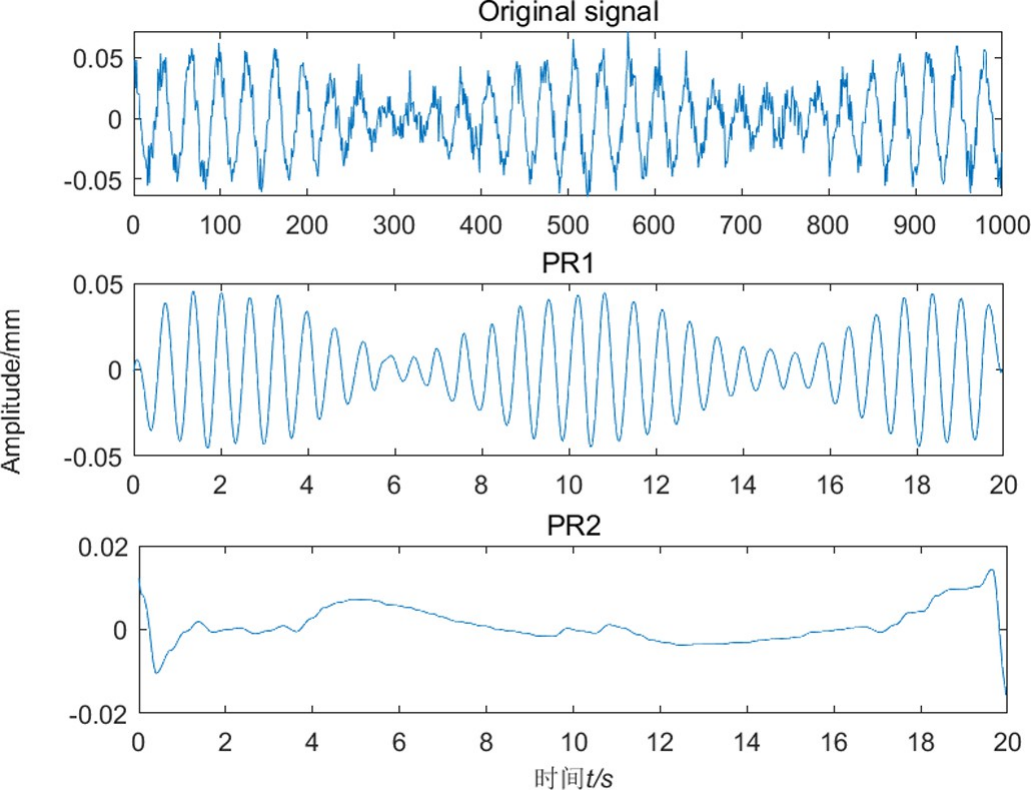
Signal decomposition by MPSO-WTD-ITD.

**Fig 8:**
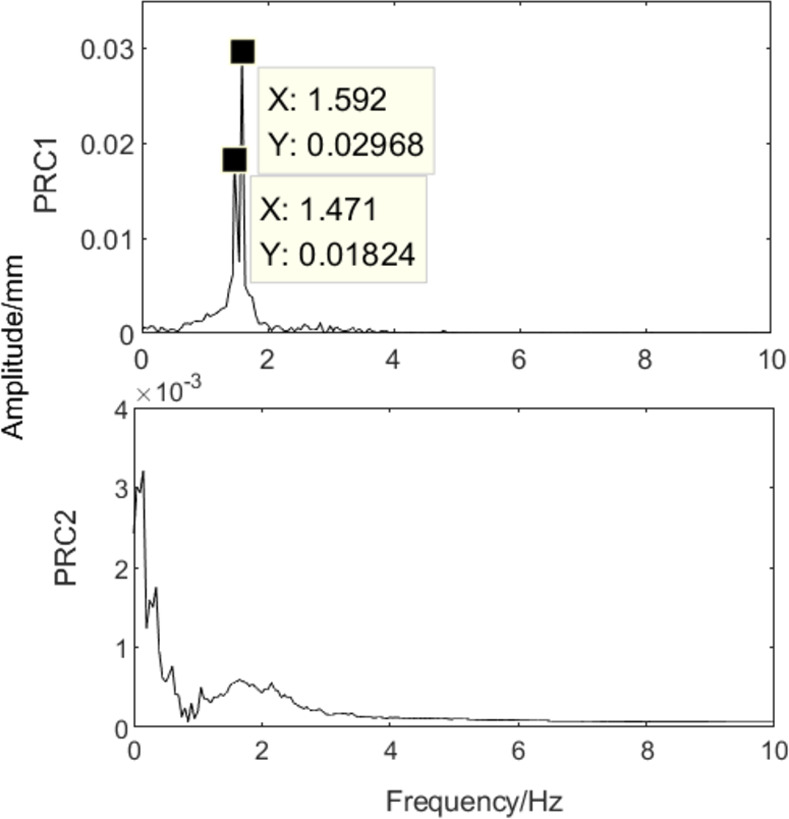
Amplitude-frequency characteristics.

The MPSO-WTD-ITD method was used to extract the roll eccentricity from the model presented in Formula (12). The results are shown in Figs 7 and 8. The eccentricity signal was decomposed into one PRC component and one residual component, as shown in Fig 7. Fourier analysis was performed on the PRC component, as shown in Fig 8. The results are consistent with those obtained by the wavelet and ITD algorithms. The proposed MPSO-WTD-ITD method can suppress spectrum aliasing and spectrum chaos phenomena in wavelet decomposition, reduce the influence of noise signals, and accurately extract the characteristic frequency of the eccentricity signal.

## 4. Experimental verification

### 4.1 Experimental setup

**Fig 9:**
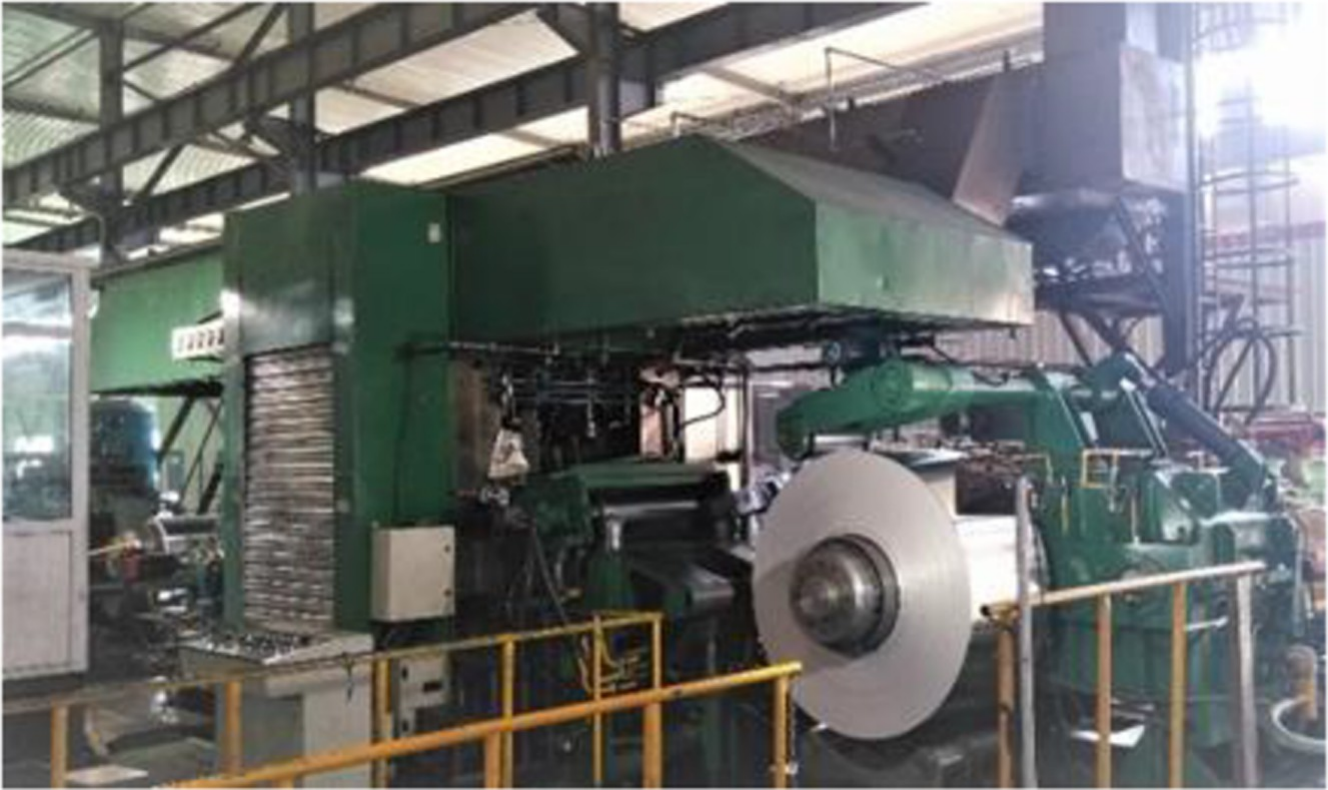
Four-high cold rolling mill.

**Fig 10:**
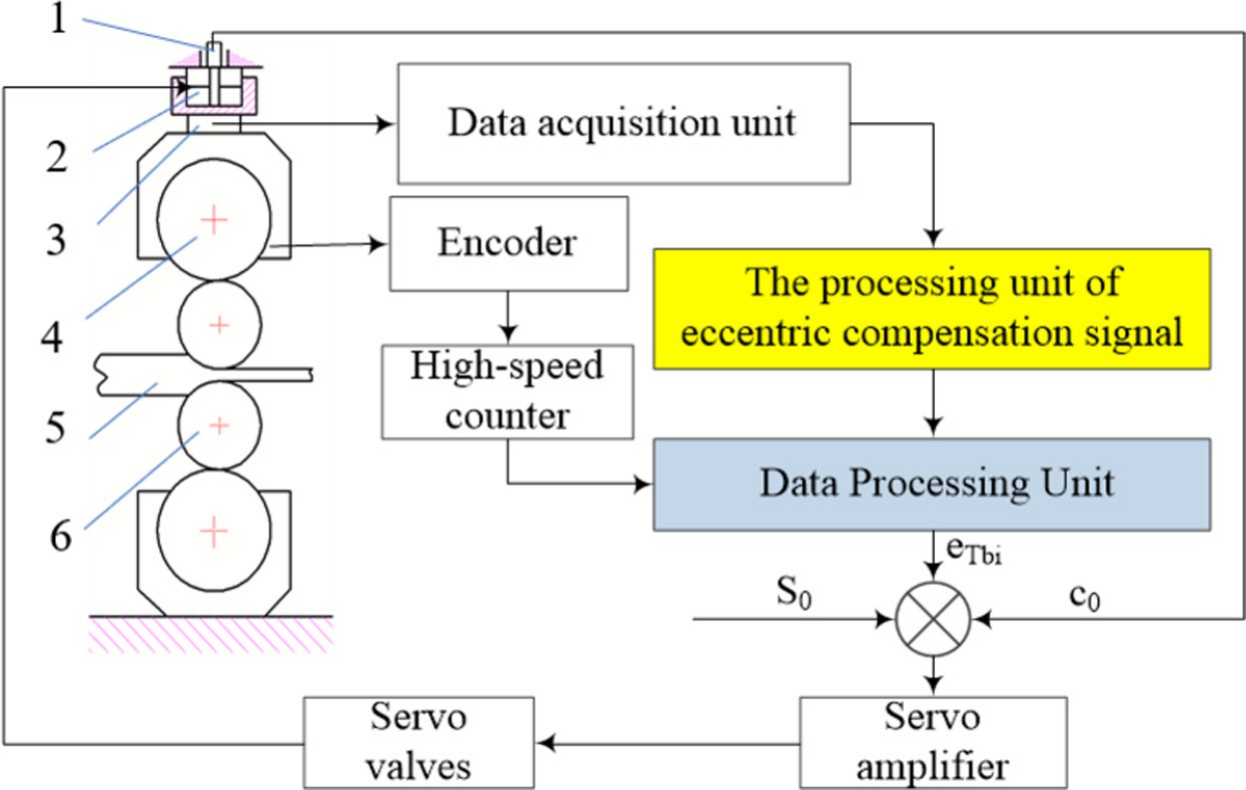
Block diagram of the control system for eccentric compensation. 1. Gap sensor 2. Hydraulic cylinder 3. Pressure sensor 4. supporting roll 5.Workpiece 6. Work roll.

A four-high cold rolling mill was used in the experiment, as shown in Fig 9. A block diagram of the test control system is presented in Fig 10. Tests were carried out using a pressure sensor, encoder, data acquisition card, and data processor. The data acquisition card was used to collect the rolling force signal measured by the pressure sensor. The processing unit of the eccentricity compensation signal was then used to obtain the roll eccentricity compensation signal. The data processing unit determines the time of sampling and the time interval for sampling according to the number of pulses obtained from the encoder. Finally, the eccentricity compensation signal, position of the hydraulic cylinder obtained by the sensor, and set value of the roll gap were determined. The output signal was amplified by a servo amplifier and sent to the servo valve to control the hydraulic cylinder.

### 4.2 Test procedure

#### 4.2.1 Extraction of roll eccentricity signal

**Fig 11:**
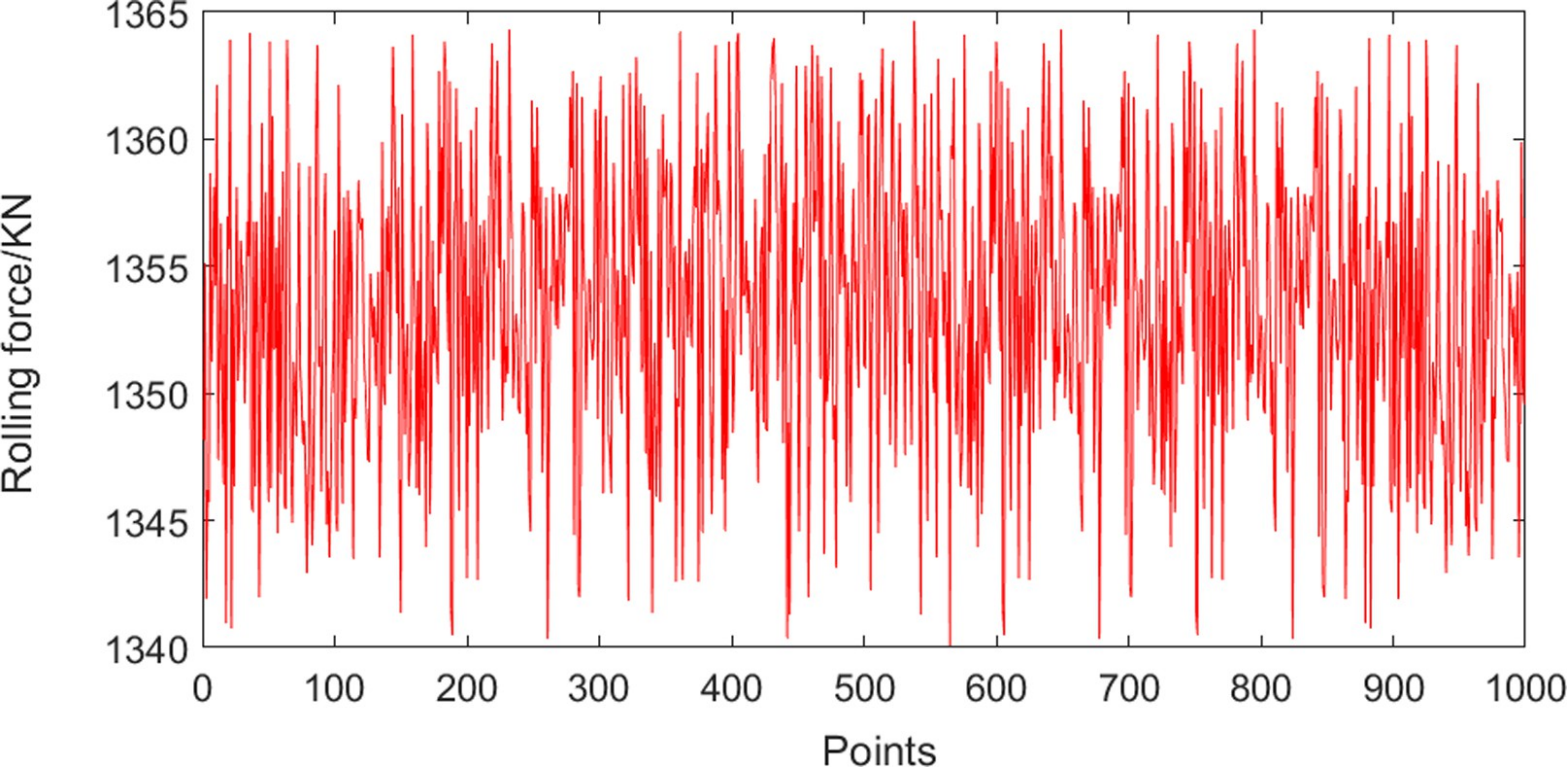
Rolling force signal.

The easiest way to measure roll eccentricity is by recording fluctuations in the rolling force under rolling mill preloading [22]. The acquisition period was 0.01 s and the rolling speed was 3.5 m/s. A representative rolling force signal is shown in Fig 11.

#### 4.2.2 Compensation of roll eccentricity

*4*.*2*.*2*.*1 Determination of signal sampling time*. To accurately calibrate the compensation signal to the corresponding position of the roll, the number of sampling points and the number of eccentricity signal compensation points should be the same after one complete rotation cycle of the roll. To prevent changes in rolling speed from affecting the accuracy of eccentricity compensation at each compensation position, an encoder was installed on the support roll. The data processing unit determines the sampling time according to the number of encoder pulses. For example, if the control cycle of the system is 0.01 s, the roll rotates once every 0.6 s; the encoder will emit 1200 pulses, and the system will collect samples at 20-pulses intervals. When the rotation time of the roll changes to 0.5 s, the system will collect samples at 24-pulses interval. The number of sampled points is the number of points compensated within the compensation period of the current eccentricity signal.

*4*.*2*.*2*.*2 Eccentricity signal conversion*. In this study, the AGC system of the cold rolling mill adopted a closed-loop position control method. The roll gap value was controlled by controlling the hydraulic cylinder displacement. Therefore, it was necessary to convert the extracted rolling force eccentricity signal △P_e_ into displacement compensation quantity *e* using the following formula:

$$e=\frac{K_{M}+K_{S}}{K_{M}K_{S}}\Delta P_{e}$$ (13)

where *K*_*M*_ is the plasticity coefficient of the rolled piece and *K*_*S*_ is the rolling mill stiffness.

**Fig 12:**
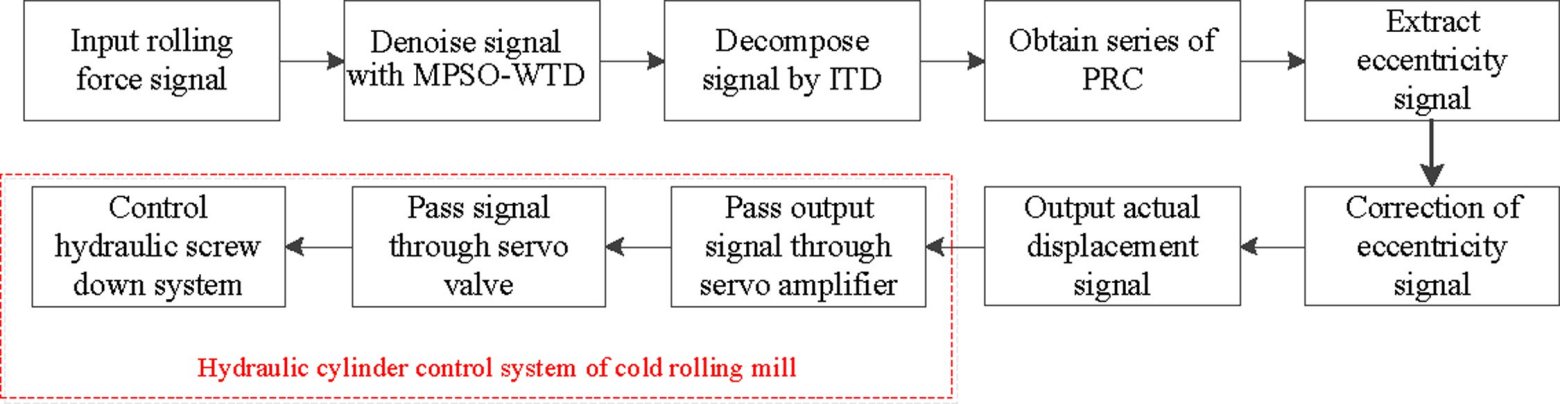
Roll eccentricity signal compensation process.

The roll eccentricity signal compensation process with MPSO-WTD-ITD is shown in Fig 12. The specific steps of the process can be summarized as follows:

1. The rolling force signal was collected from the rolling mill under the no-load condition. The rolling thickness, fluctuation in hardness, and other factors were not considered. The signal was preprocessed with MPSO-WTD for denoising.
2. The ITD method was used to extract the rolling force eccentricity signal.
3. According to Eq (13), the extracted rolling force eccentricity signal was converted into the displacement compensation signal.
4. The compensation signal was input into the AGC system of the rolling mill, and a point-to-point eccentricity signal of the roll was realized according to the number of signal pulses output by the encoder.
5. The output displacement was obtained according to the compensation signal, set value of the roll gap, and actual position of the hydraulic cylinder, and the position of the hydraulic cylinder of the hydraulic press was controlled by the servo amplifier and electro-hydraulic servo valve.

**Fig 13:**
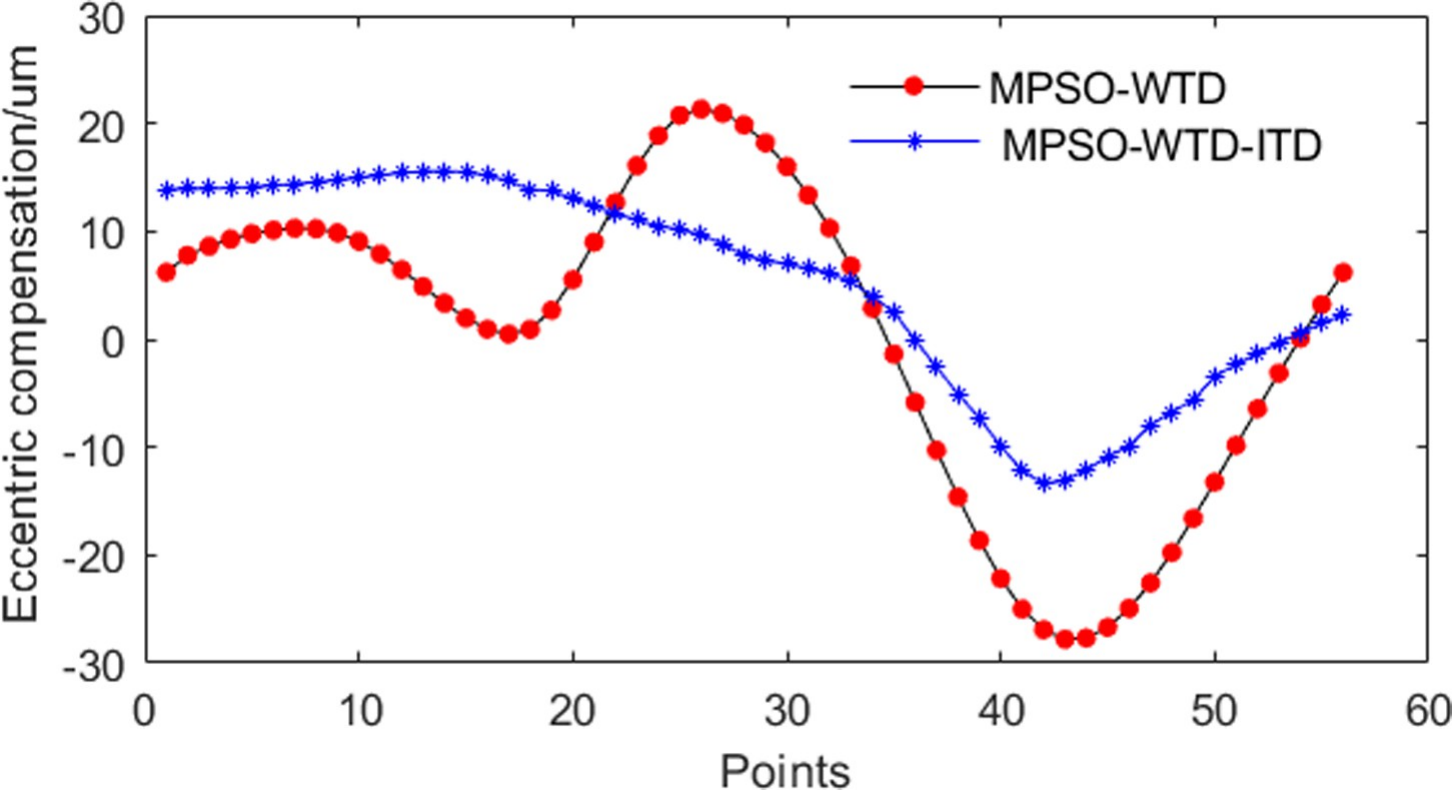
Eccentric compensation signal.

As shown in Fig 13, the roll eccentricity compensation signal was obtained using the wavelet algorithm and MPSO algorithm, and can be divided into 56 equally spaced points.

### 4.3 Analysis of test results

The controlled plant in the experiment was a four-high cold rolling mill. The work roll diameter was 300 mm, and the backup roll diameter was 600 mm. The rolling speed was 3.5 m/s. The entrance thickness of the aluminum alloy 3004 strip was 1 mm, and the outlet thickness set-point was 0.52 mm. During the experiment, the rolling force data were updated with the newly sampled data in each control step.

**Fig 14:**
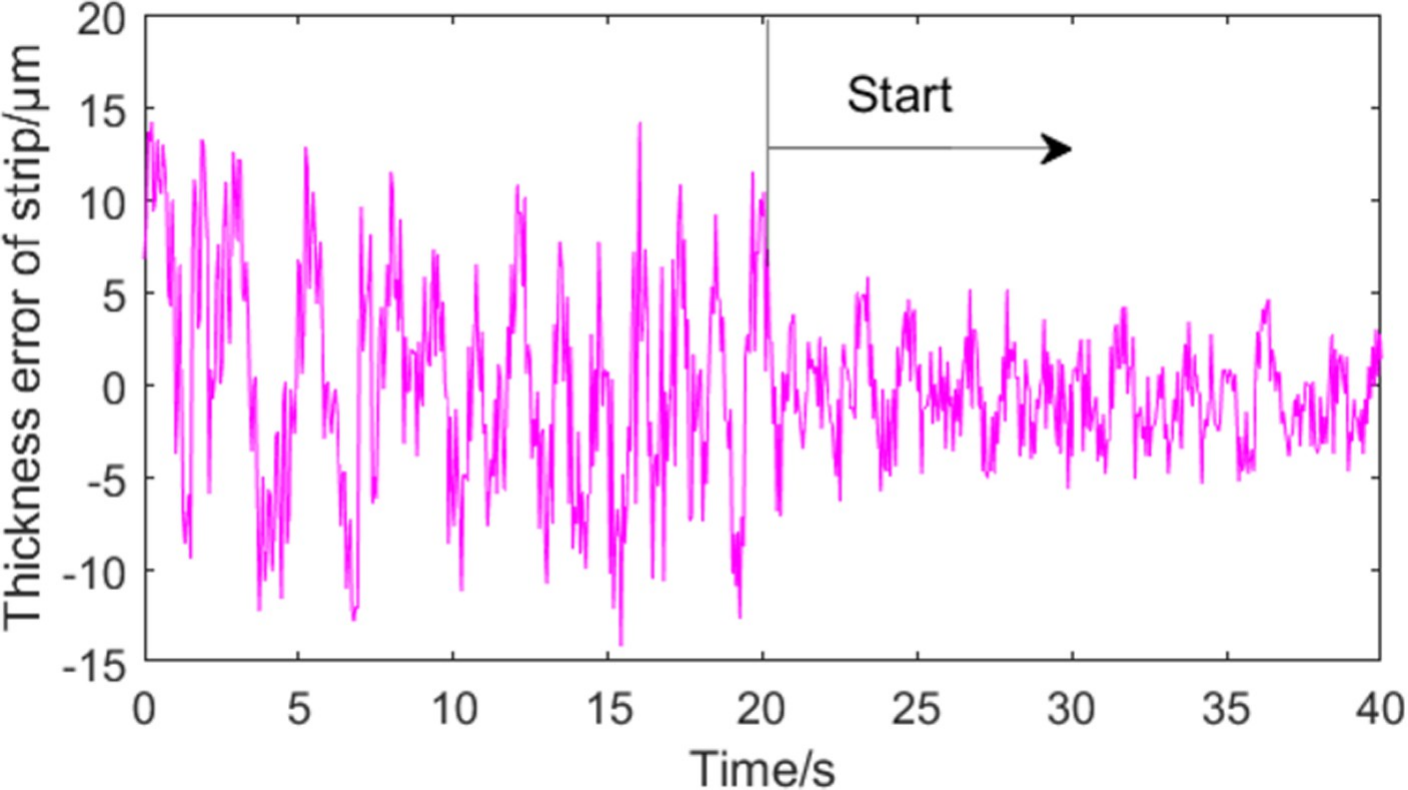
Effect of eccentricity compensation on strip thickness in accelerated rolling stage.

**Fig 15:**
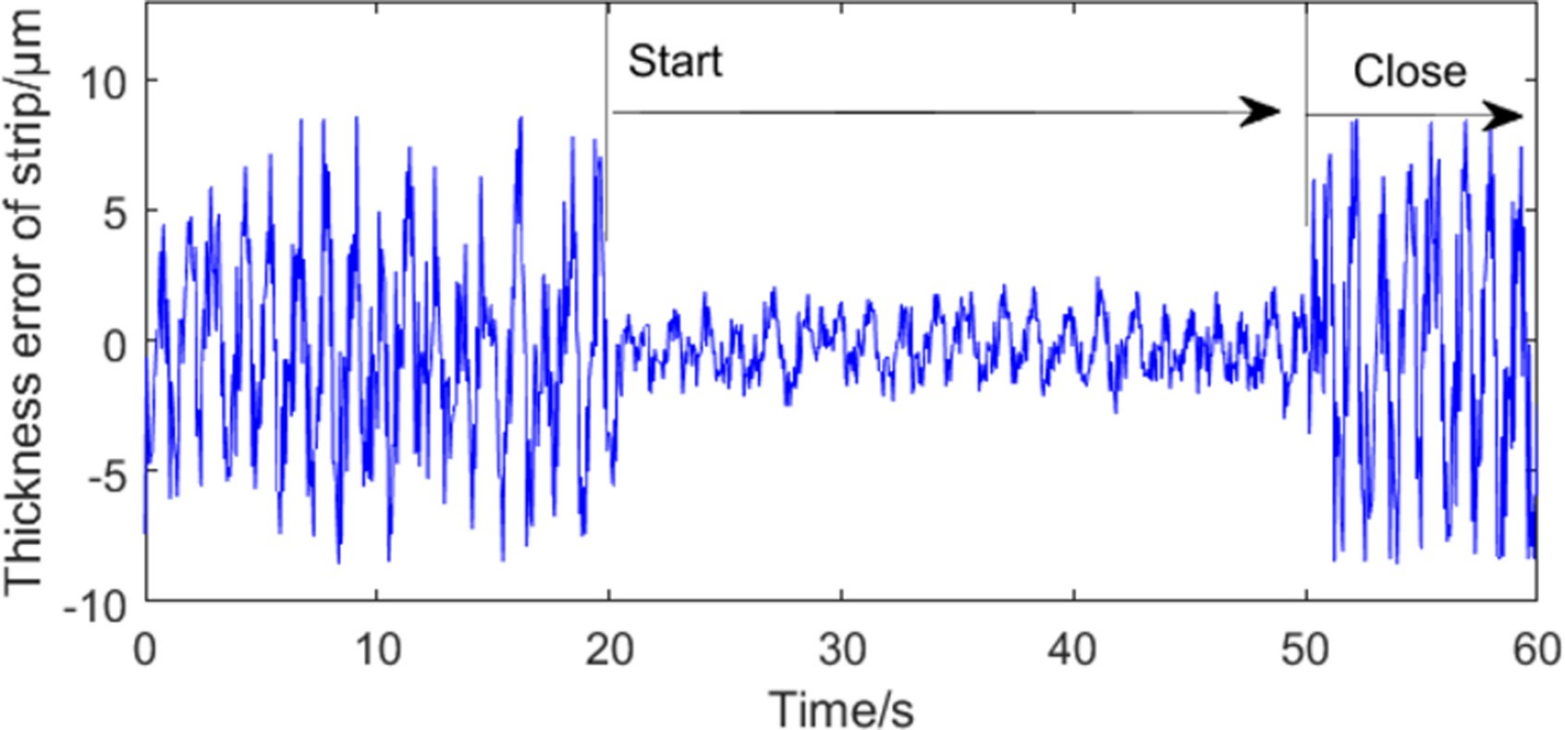
Effect of eccentricity compensation on strip thickness in stable rolling stage.

Figs 14 and 15 show the control effect of roll eccentricity compensation on strip thickness using the MPSO-WTD-ITD method. Fig 14 shows the control effect on strip thickness in the increasing rolling speed stage. Fig 15 shows the control effect on strip thickness in the stable rolling stage. The experimental results suggest that roll eccentricity compensation effect is better during the stable rolling stage. Strip thickness compensation can be achieved even when the rolling speed is not uniform due to high precision of the roll eccentricity signal extraction process and control system.

The roll eccentricity compensation effect can be expressed as the rate of improvement in strip thickness characteristic R using the following formula:

$$R=\frac{H_{e}-H}{H}$$ (14)

where *H*_*e*_ is range of strip thickness fluctuation when roll eccentricity is controlled and H is the range of strip thickness fluctuation when roll eccentricity is not controlled.

The eccentricity compensation achieved by the two methods was input into the automatic gauge control (AGC) system of the cold rolling mill. The effects of roll eccentricity compensation in the stable rolling stage are presented in Table 1. A total of 100 strip thickness values were collected for normal distribution statistical analysis. It can be concluded that the probability of a strip thickness error of ±3.5μm is about 96%, and the roll eccentricity compensation effect reaches 62.3% during steady-state rolling.

**Table 1:** Effects of roll eccentricity compensation in stable rolling stage.

Thickness of end rolling strip (mm)	Variation of strip thickness at outlet (μm) (Finish rolling)			Improvement strip thickness characteristic (R)	
	Without roll eccentricity control	Roll eccentricity control (wavelet)	Roll eccentricity control (MPSO-WTD-ITD)	Wavelet	MPSO-WTD-ITD
**0.52**	±9.3	±4.5	±3.5	51.6%	62.3%

## 5. Conclusions

A roll eccentricity extraction method based on MPSO-TWD and ITD was proposed to improve the accuracy of roll eccentricity signal extraction. Simulations were carried out the roll eccentricity compensation signal and were also input into the AGC system of a four-high cold rolling mill to verify the results. The main conclusions of this study can be summarized as follows:

1. The gradient iteration method and MPSO algorithm were used to calculate the threshold and coefficient of wavelet, respectively. The simulation results show that the MPSO-WTD method has better denoising effects. Comparing the denoising effects of ITD, MPSO-WTD, and MPSO-WTD-ITD on roller eccentricity signals, the simulation results show that the proposed method avoids the frequency aliasing phenomenon of wavelet analysis and poor anti-noise performance of the ITD method. The proposed method has high precision in extracting roller eccentricity signals.
2. To avoid the influence of rolling speed on the frequency of the roll eccentricity signal, the frequency of the eccentricity compensation signal is determined according to the number of encoder pulses. Due to the complexity of the rolling process and accuracy limitations of the test equipment, the strip thickness control will be affected. However, the experimental results show that the proposed method is satisfactory. It can be concluded that 96% of the strip thickness errors can be controlled to ±3.5μm and the roll eccentricity compensation effect reaches 62.3% during steady-state rolling by collected 100 strip thickness.

## Supporting information

S1 Data (XLSX)
